# A Review of SARS-CoV-2 and the Ongoing Clinical Trials

**DOI:** 10.3390/ijms21072657

**Published:** 2020-04-10

**Authors:** Yung-Fang Tu, Chian-Shiu Chien, Aliaksandr A. Yarmishyn, Yi-Ying Lin, Yung-Hung Luo, Yi-Tsung Lin, Wei-Yi Lai, De-Ming Yang, Shih-Jie Chou, Yi-Ping Yang, Mong-Lien Wang, Shih-Hwa Chiou

**Affiliations:** 1 Department of Medical Research, Taipei Veterans General Hospital, Taipei 11217, Taiwan; tina122143@gmail.com (Y.-F.T.); polo661124@yahoo.com.tw (C.-S.C.); yarmishyn@gmail.com (A.A.Y.); s19609005@gm.ym.edu.tw (Y.-Y.L.); jefflai8228@gmail.com (W.-Y.L.); yang.deming@gmail.com (D.-M.Y.); ohyeahchou@gmail.com (S.-J.C.); d49624011@gm.ym.edu.tw (Y.-P.Y.); 2 School of Medicine, National Yang-Ming University, Taipei 11221, Taiwan; hecterlo@gmail.com (Y.-H.L.); ytlin8@vghtpe.gov.tw (Y.-T.L.); 3 Institute of Pharmacology, National Yang-Ming University, Taipei 11221, Taiwan; 4 Department of Chest Medicine, Taipei Veterans General Hospital, Taipei 11217, Taiwan; 5 Division of Infectious Diseases, Department of Medicine, Taipei Veterans General Hospital, Taipei 11217, Taiwan; 6 Institute of Food Safety and Health Risk Assessment, School of Pharmaceutical Sciences, National Yang Ming University, Taipei 11221, Taiwan; 7 Genomic Research Center, Academia Sinica, Taipei 11529, Taiwan

**Keywords:** SARS-CoV-2, COVID-19, pneumonia, clinical trials, immunotherapy, vaccine, ACE2, replicase

## Abstract

The sudden outbreak of 2019 novel coronavirus (2019-nCoV, later named SARS-CoV-2) in Wuhan, China, which rapidly grew into a global pandemic, marked the third introduction of a virulent coronavirus into the human society, affecting not only the healthcare system, but also the global economy. Although our understanding of coronaviruses has undergone a huge leap after two precedents, the effective approaches to treatment and epidemiological control are still lacking. In this article, we present a succinct overview of the epidemiology, clinical features, and molecular characteristics of SARS-CoV-2. We summarize the current epidemiological and clinical data from the initial Wuhan studies, and emphasize several features of SARS-CoV-2, which differentiate it from SARS-CoV and Middle East respiratory syndrome coronavirus (MERS-CoV), such as high variability of disease presentation. We systematize the current clinical trials that have been rapidly initiated after the outbreak of COVID-19 pandemic. Whereas the trials on SARS-CoV-2 genome-based specific vaccines and therapeutic antibodies are currently being tested, this solution is more long-term, as they require thorough testing of their safety. On the other hand, the repurposing of the existing therapeutic agents previously designed for other virus infections and pathologies happens to be the only practical approach as a rapid response measure to the emergent pandemic, as most of these agents have already been tested for their safety. These agents can be divided into two broad categories, those that can directly target the virus replication cycle, and those based on immunotherapy approaches either aimed to boost innate antiviral immune responses or alleviate damage induced by dysregulated inflammatory responses. The initial clinical studies revealed the promising therapeutic potential of several of such drugs, including *favipiravir*, a broad-spectrum antiviral drug that interferes with the viral replication, and *hydroxychloroquine*, the repurposed antimalarial drug that interferes with the virus endosomal entry pathway. We speculate that the current pandemic emergency will be a trigger for more systematic drug repurposing design approaches based on big data analysis.

## 1. Introduction

The twenty-first century has experienced the emergence and epidemic of three previously unidentified coronaviruses: severe acute respiratory syndrome coronavirus (SARS-CoV) in 2003, Middle East respiratory syndrome coronavirus (MERS-CoV) in 2012, and 2019 novel coronavirus (2019-nCoV, later officially named SARS-CoV-2) in late December, 2019. All of them belong to the Coronaviridae, a family of viruses that possess a positive-sense single-stranded RNA genome. Similar to other RNA viruses, this family is characterized by significant genetic variability and high recombination rate that enable them to be easily distributed among humans and animals worldwide. As a result, numerous coronaviruses exist within human and animal populations without causing life-threatening diseases. However, occasionally, the genetic recombination of viruses within random intermediate hosts produces contagious strains that are highly pathogenic to humans. Whereas SARS-CoV-2 is genetically and structurally related to SARS-CoV, it is becoming increasingly clear that it has its own unique features that contributed to the rapid spread around the globe.

Since there is currently no effective treatment available for coronavirus infections, significant efforts have been made to the development of vaccines and therapeutic drugs. Preclinical evidence has proven the potential of several countermeasures, yet large scale trials are still needed. Here, we review the current understanding of SARS-CoV-2 such as its epidemiological and clinical features, as well as its unique contagious characteristics different from SARS-CoV and MERS-CoV, providing the essential information for adjusting our responses against the SARS-CoV-2 pandemic. We also summarize the state-of-the-art innovations on targeting SARS-CoV-2 through a biological and pathological point of view.

## 2. Epidemiology

As of April 6, 2020, a total of 1,285,257 cases of COVID-19 occurring in at least 170 countries and territories were reported, with approximately 5.4 % of fatality rate (70,344/1,285,257). A prior overview from China that included 72,314 confirmed, suspected, and asymptomatic patients revealed several important epidemiological features of COVID-19. In general, the majority of confirmed cases are aged 30–79 years-old (86.6%) [1]. Among the 1,023 deaths, the majority were among patients of ≥60 years of age, with the ≥80 age group characterized by the highest fatality rate (20.3%) among all age groups. Ref. [1] Relatively fewer cases were reported among young children (0-9 years-old). Ref. [1] While more males were affected by the disease, the male-to-female ratio varies when focusing on different population scales (1.06:1 in China overall, 1.04:1 in Hubei province and 0.99:1 in Wuhan city). Fortunately, for the most affected patients, COVID-19 pneumonia presentation has been mild. Moreover, while this pathogen has been extraordinarily contagious, no deaths have occurred in mild or even severe cases. As to be expected, the fatality rate reached 49% among patients that were classified as critical cases.

## 3. General Clinical Features of COVID-19

The usual symptoms of COVID-19 include fever (83–98%), cough (59–82%), shortness of breath (19–55%), and muscle ache (11–44%), which are similar to those of SARS and MERS. Ref. [2] Some patients may have sore throat, rhinorrhea, headache and confusion a few days before the onset of fever, indicating that fever is a critical symptom, but not the only initial manifestation of infection. Ref. [2] The pattern of fever has not yet been fully understood. A small proportion of patients had hemoptysis [3,4], and a number of cases were found relatively asymptomatic. Ref. [5] COVID-19 patients may have normal or lower white blood cell counts, lymphopenia, or thrombocytopenia, with the increased C-reactive protein level. Ref. [2,3,4] People who have fever and upper respiratory tract symptoms with leukopenia or lymphopenia should be suspected for this disease, especially for patients with travel history to the endemic area or close exposure record.

However, the clinical course of COVID-19 pneumonia exhibits a broad spectrum of severity and progression patterns. In some patients, dyspnea develops within a median of 8 days after the onset of illness (range of 5–13 days), while in others, respiratory distress may be absent. Ref. [3] Around 3–29% patients may need the admission to the intensive care unit. Severely ill patients may have poor disease course of rapid progression to multiple organ dysfunction and even death [2,3], and those who have shortness of breath and hypoxemia can quickly progress into acute respiratory distress syndrome (ARDS), severe sepsis with shock, and even multiple organ dysfunction within one week. Ref. [4,6] ARDS was observed to develop in 17–29% of hospitalized patients approximately 8 days after symptoms onset, and the global mortality rate reached approximately 5.4% [3].

It is also worth noting that the gastrointestinal symptoms of COVID-19 may be caused by the direct viral damage to the intestine rather than the immunopathogenic response to the lung infection of the host. Since angiotensin-converting enzyme 2 (ACE2), the main cellular receptor of SARS-CoV-2 is expressed in the human gastrointestinal epithelial cells, it is believed that the viral shedding at the gastrointestinal tract and fecal–oral transmission is highly plausible. Ref. [7] Indeed, it was reported that the rectal swabs showed positive results even after the nasopharyngeal tests were constitutively negative [8]. Besides, the live virus was also detected in stool samples of diseased patients. This evidence strongly indicate that stool can be contagious for a long time after the discharge of patients based on two negative nasopharyngeal swabs. Thus, adding rectal swabs to the discharge criteria should be considered for the prevention of both nosocomial and community spread of COVID-19.

Aside from the gastrointestinal symptoms, a retrospective study of 214 patients in China reported that 5.6 % of patients experienced hypogeusia and 5.1 % experienced hyposmia [9]. Though the loss of olfaction during SARS-CoV-2 infection could be explained by the swelling of the nasal mucosa, a larger population of patients should be included to determine whether hypogeusia and hyposmia could be a common neurological manifestation of COVID-19. Nevertheless, hyposmia and hypogeusia are now being recommended as the early warning signs and an indication for early self-isolation.

## 4. Radiological Features of COVID-19

The radiological examinations, including chest X ray (CXR) and chest computed tomography (CT) scan, are important for early detection and treatment of COVID-19 [10]. The imaging findings of COVID-19 pneumonia mimic influenza, SARS-CoV, and MERS-CoV pneumonia [11,12,13,14,15]. The primary Wuhan study revealed that upon diagnosis, 74 [75%] patients showed bilateral pneumonia, and the remaining 25 [25%] patients showed unilateral pneumonia. Ref. [2] In addition, 14 [14%] patients showed multiple mottling and ground-glass opacities [2]. In the subsequent study, it was reported that the predominant pattern of abnormality observed was peripheral (44 [54%]), ill-defined (66 [81%]), and mainly involved the right lower lobes (225 [27%] of 849 affected segments) [2]. Bilateral multiple consolidation usually occurs in more severe cases [10].

Chest CT is more efficient in detecting pneumonia at the early stages of COVID-19. However, the imaging findings of COVID-19 pneumonia on chest CT are variable and nonspecific [16,17,18]. The most common patterns of COVID-19 on chest CT scans include multiple GGO lesions (56.4%), and bilateral patchy shadowing (51.8%), and the other patterns consist of local patchy shadowing (28.1%), and interstitial abnormalities (4.4%). Severe cases tend to yield more prominent radiologic findings on chest CT scan, such as more bilateral patchy shadowing (82%), more multiple GGO lesions (60%), and more local patchy shadowing (55.1%) than non-severe cases. No CXR or chest CT abnormality was identified in 17.9% of non-severe cases and 2.9% of severe cases [1,2,3]. Pure GGO lesions can be found in the early stages. Focal or multifocal GGO lesions may progress into consolidation or GGO lesions with superimposed interlobular/intralobular septal thickening as crazy-paving pattern during disease progression, and the expansion of consolidation represented disease progression [19,20,21]. Pure consolidative lesions were relatively less common. Pulmonary cavitary lesion, pleural effusion, and lymphadenopathy are rarely reported [19,20,21,22].

However, interestingly, it was also reported that asymptomatic patients could show early CT changes [23]. Conversely, as mentioned earlier, another study has shown positive RT-PCR results for SARS-CoV-2 in the absence of CT changes [24]. Despite the limited number of cases available for thorough radiographic study, we can observe the trend of varied presentations of COVID-19 pneumonia. Asymptomatic patients showing positive CT findings undoubtedly pose challenges for the current diagnostic protocol, especially those patients who have false-negative RT-PCR results.

**Figure 1:**
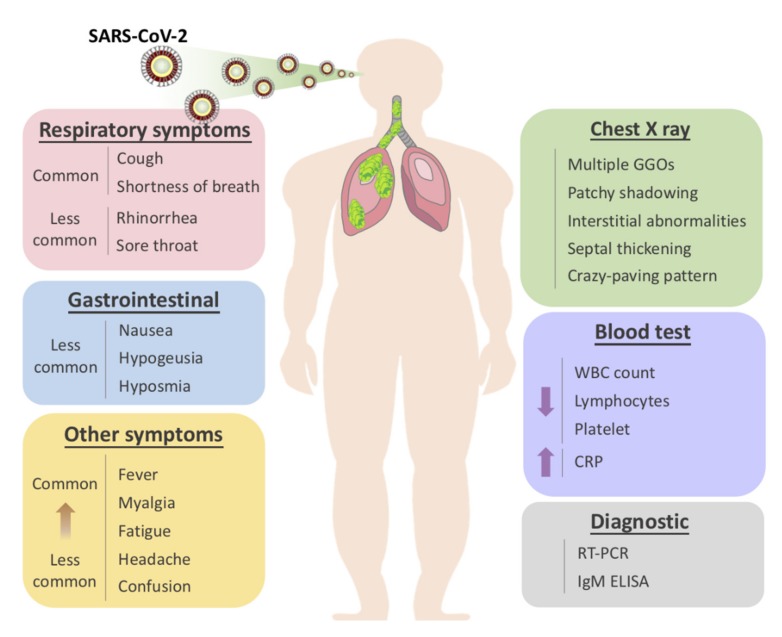
Overview of symptomatic, radiological and laboratory characteristics of COVID-19.

Moreover, different radiographic patterns are seen as the COVID-19 progresses. Typically, after the first to second week of the onset, lesions progress to bilateral diffused pattern with consolidations. By contrast, both ground-glass opacification and consolidation were present relatively early in SARS [6]. This again could be indicative of the significant difference in diagnostic sensitivity between these two diseases, especially at early or asymptomatic stage. In conclusion, correlating imaging features with clinical and laboratory findings to assess patients may be essential to facilitate early diagnosis of COVID-19 pneumonia (Figure 1).

### Comparison between Severe Acute Respiratory Syndrome Coronavirus (SARS-CoV), Middle East Respiratory Syndrome Coronavirus (MERS-CoV), and SARS-CoV-2

Whereas several human coronaviruses that cause mild respiratory diseases, such as HCoV-229E, HCoV-OC43, HCoV-NL63, and HCoV-HKU1, were estimated to circulate in the human population for centuries, SARS-CoV, MERS-CoV, and SARS-CoV-2 were zoonotically transferred from other mammalian species in the last 20 years [12,13,14,15]. Horseshoe bats are the natural reservoirs of these novel coronaviruses, and the intermediate hosts that transmitted the virus to the human were identified to be the masked palm civet for SARS-CoV, and dromedary camel for MERS-CoV (Table 1). The recent metagenomics study has detected the most similar coronaviruses to SARS-CoV-2 in the Malayan pangolin (*Manis javanica*), one of the species presumably smuggled to the Huanan wet market in Wuhan [25].

The difference in the transmission patterns between SARS-CoV, MERS-CoV, and SARS-CoV-2 is also indicative of the specific intrinsic characteristics of SARS-CoV-2 [12]. In the case of SARS-CoV and MERS-CoV, substantial virus shedding happens only after the onset of symptoms, therefore, the transmission mainly occurs in a nosocomial manner, namely, after the infected patients have sought medical help [35]. However, human-to-human transmission of SARS-CoV-2 occurs predominantly in communities and between family members, which might indicate that the pathogen could be spread far before the onset of symptoms. A recent study suggested that the half-lives of SARS-CoV-2 and SARS-CoV were similar in aerosols with the median infectious period estimated to be around 1.1 to 1.2 hour [27]. Therefore, as an echo to SARS-CoV, the possibility of air-borne and fecal–oral transmission of SARS-CoV-2 cannot be ruled out, however, more evidence is still needed.

In addition to the pre-existing factors that contribute to the blind spot of disease control, previous studies found that during active surveillance, two individuals with close contact history with confirmed cases showed positive results on RT-PCR. Another report revealed patients that had been proven to recover from COVID-19 by two consecutive RT-PCR tests, turned out to show positive results a few days later. While the patients continued to be asymptomatic and no people within their close contact were infected, they were still considered as infectious viral carriers [24].

In conclusion, the evidence exists that the infected cases can be contagious before the onset and after treatment of COVID-19 pneumonia. Thus, current criteria for hospital discharge and discontinuation of quarantine may have to be reevaluated in order to achieve a more intact protocol for adequate disease control. Table 1 compares different features of SARS-CoV, MERS-CoV, and SARS-CoV-2.

## 5. Structure and Genomic Characteristics

SARS-CoV, MERS-CoV, and SARS-CoV-2 belong to the Coronaviridae family. This family of viruses contains a relatively large single-stranded, positive-sense RNA genome of around 27–32 kb. Ref. [36] Their genomes are typically composed of a 5′-methylguanosine cap at the beginning, a 3′-poly-A tail at the end, and a total of 6-10 genes in between [12] The order of their genes is usually highly conserved, with the first one being replication- and transcription-related, and the rest, structural. The replication- and transcription-related gene is translated into two large non-structural polyproteins by two open reading frames. Ref. [12] The two different yet overlapping open reading frames are translated by ribosomal frameshifting. On the other hand, the structural proteins, including the spike (S), envelope (E), and membrane (M) that constitute the viral coat, and the nucleocapsid (N) protein that packages the viral genome, are translated from the subgenomic RNAs. Some of these proteins undergo glycosylation in the Golgi apparatus to form glycoproteins.

Among all the structural proteins, the most important potential therapeutic target is the spike (S) glycoprotein, which is responsible for the binding of the virus to the host cells. S protein is primed by the host cell protease and is recognized by the cellular receptor. Ref. [37] The human serine protease TMPRSS2 is responsible for priming the S protein of both SARS-CoV and SARS-CoV-2, and the angiotensin-converting enzyme 2 (ACE2) is engaged as a receptor for the entry of these two viruses. As for MERS-CoV, it binds specifically to another receptor, dipeptidyl peptidase 4 (DPP4) [12].

It is worth noting that not only ACE2 expression level, but also the allele frequency varies among populations. As several ACE2 variants have been identified, the correlation between disease susceptibility and sequence polymorphism has been hypothesized. However, the previous study on confirmed cases found no heterogeneity among the residues implicated in the viral S protein binding, indicating that SARS-CoV-2 may associate with a highly-conserved site of human genome. The indistinguishable susceptibility between individuals can be another reason for the rapid spread of SARS-CoV-2 across continents and different human populations [38].

The characteristics of the cellular receptor ACE2 can also explain the pathogenesis features of SARS-CoV and SARS-CoV-2. It has been reported that the binding of the viral S protein to ACE2 induces a negative feedback loop that ultimately results in downregulation of ACE2. The decrease of ACE2 subsequently directs its substrate angiotensin I towards its related enzyme, ACE. Increased ACE activity consequently results in the elevated levels of angiotensin II. Once angiotensin II binds to its receptor, AGTR1A, pulmonary vascular permeability is increased [39].

## 6. Current Treatment Modalities

### 6.1. Diagnostics

It is recommended by the Centers of Disease Control and Prevention that health care professionals obtain specimens from both upper respiratory tract (either nasopharyngeal or oropharyngeal) and lower respiratory tract (either endotracheal tube or bronchoalveolar lavage). Diagnosis of COVID-19 pneumonia is primarily based on the RT-PCR analysis of the specimens. If RT-PCR is unavailable, serology test may also be considered.

Currently, the U.S. Food and Drug Administration (FDA) has approved a SARS-CoV-2 commercial test system from Roche (cobas^®^ SARS-CoV-2). This qualitative test requires samples from nasopharyngeal or oropharyngeal swabs, and it takes 3.5 h to yield the results. Based on RT-PCR methodology, the cobas SARS-CoV-2 test is a dual target assay, detecting both the specific SARS-CoV-2 RNA, as well as the highly conserved fragment of the E gene invariant in all members of the *Sarbecovirus* subgenus. The assay has a full-process negative control, positive control and internal control to ensure specificity and accuracy. On 21 March 2020, FDA granted another Emergency Use Authorization to Xpert^®^ Xpress SARS-CoV-2 from Cepheid Inc (USA), which is also a qualitative test that claimed to yield the results within 45 min. It can utilize samples from nasopharyngeal swabs, nasal wash, or aspirate specimens and highlights a hands-off, automated sample processing. The results should be viewed as positive if more than one targeted gene is present detected.

While the current screening methods rely on the presence of abundant viral genome at the site of sample collection, studies have demonstrated that the levels of IgM antibodies were high in both symptomatic and subclinical patients 5 days after onset of illness. Thus, it was proposed that IgM ELISA assay can be combined with PCR to enhance the detection sensitivity [40].

### 6.2. Therapeutics

As there is currently no specific treatment for COVID-19 pneumonia, clinical management emphasizes the importance of supportive care and prevention of complications and nosocomial transmission. When patients experience respiratory distress, oxygen should be given immediately. However, if there is no sign of tissue hypoperfusion, fluid resuscitation should be relatively conservative, as it may result in lung edema and worsen the oxygen status. This concept is particularly important in the treatment of severe acute respiratory infections [41], as it could shorten the duration of ventilation. Systemic corticosteroids are not recommended either, considering their potential to delay viral clearance. Nevertheless, exceptions could be made if corticosteroids are indicated for other reasons.

### 6.3. Precautions

Standard precautions, including respiratory and eye protection, are recommended for all healthcare professionals caring for patients with known or suspected COVID-19 pneumonia. Removal of droplet precautions can only be considered when two consecutive RT-PCR that are obtained at least 24 h apart from a clinically recovered patient both show negative results. However, based on the previous discussion, even after two sets of negative tests, there are still possibilities that the patients become viral carriers later on. We thereby suggest that the decision to remove precautions should be based not only on laboratory, radiological, and clinical evidence, but also on the professional assessment by clinicians and other specialized healthcare personnel.

## 7. Ongoing Clinical Trials

**Figure 2:**
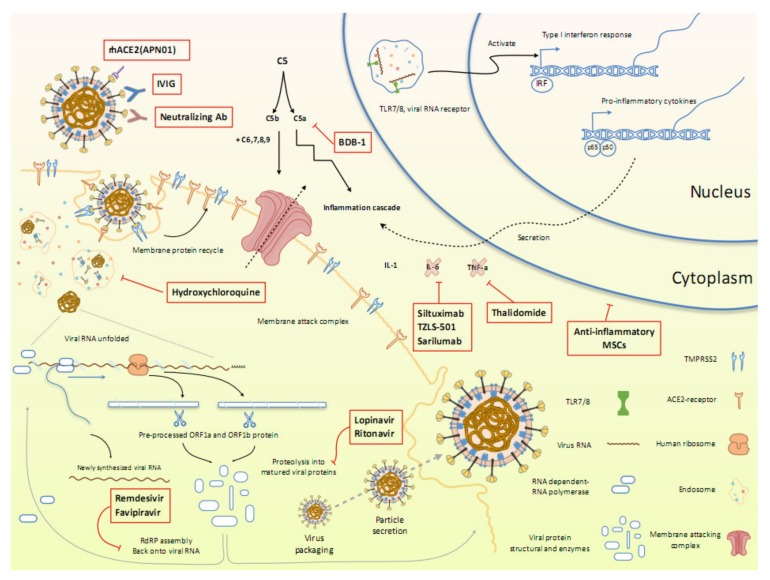
Overview of the repurposed therapeutic drugs undergoing clinical trial against COVID-19 in the context of host pathways and virus replication mechanisms.

Currently, there is not sufficient evidence that any existing antiviral drugs can efficiently treat COVID-19 pneumonia. However, there are several clinical trials on potential antiviral therapies taking place. The therapies can be divided into two categories depending on their target. One is acting on the coronavirus directly, either by inhibiting crucial viral enzyme responsible for genome replication, or by blocking viral entry to human cells. The other is designed to modulate the human immune system, either by boosting the innate response, which has a particularly important role against viruses, or by inhibiting the inflammatory processes that cause lung injury. Most of these drugs were originally designed for other pathogens and were promptly repurposed for the current COVID-19 trials. At the same time, several trials were initiated to test the specific vaccines and antibodies specifically targeting SARS-CoV-2. Here, we summarize the ongoing therapeutic options that may lead us to combating the novel pathogen (Figure 2).

### 7.1. Inhibiting the RNA-dependent RNA polymerase

#### 7.1.1. Remdesivir

Remdesivir (GS-5734) is by far the most promising drug that exhibits broad-spectrum antiviral activities against RNA viruses. It is a prodrug, whose structure resembles adenosine. Ref. [42] Therefore, it can incorporate into nascent viral RNA, and further inhibit the RNA-dependent RNA polymerase. This results in premature termination of the viral RNA chain and consequently halts the replication of the viral genome. Remdesivir was originally developed by Gilead Sciences (USA) against the Ebola virus, and has undergone clinical trial during the recent Ebola outbreak in the Democratic Republic of Congo [43]. Although it has not been shown to be effective against Ebola in this trial, it proved its safety for humans, which allowed it to enter clinical trials immediately in the conditions of COVID-19 emergency [43]. Importantly, it has been previously shown to exhibit antiviral activities against different coronaviruses, including SARS-CoV and MERS-CoV, in vitro and in vivo [44,45]. In a recent in vitro study, remdesivir was also shown to inhibit SARS-CoV-2 [46].

As was reported in the case report of the first COVID-19 patient in the USA, remdesivir was used on the 7th day of hospitalization without any noticeable adverse effect, and the patient’s condition improved on the 8th day [47]. Remdesivir is now being tested in multiple trials in different countries, including two randomized phase III trials in China (NCT04252664 and NCT04257656) that are expected to be completed in April/May 2020.

#### 7.1.2. Favipiravir

Similar to remdesivir, favipiravir, developed by Toyama Chemical (division of Fujifilm, Japan), functions as an inhibitor of the RNA-dependent RNA polymerase by structurally resembling the endogenous guanine [48]. Through competitive inhibition, the efficacy of viral replication can be hugely reduced. Although it is an approved treatment for influenza, less preclinical support has been established for favipiravir to treat SARS-CoV-2 compared to remdesivir. Nevertheless, patients have been recruited to evaluate the efficacy of favipiravir plus interferon-α (ChiCTR2000029600) based on the expected synergistic effect of viral inhibition and immune enhancement. Indeed, in March 2020, favipiravir was approved by the National Medical Products Administration of China as the first anti-COVID-19 drug in China, as the clinical trial had demonstrated efficacy with minimal side effects.

### 7.2. Inhibiting the Viral Protease

#### 7.2.1. Ivermectin

Ivermectin is an FDA-approved anti-parasitic agent which was also proven to exert antiviral activities toward both human immunodeficiency virus (HIV) and dengue virus [49].

It can dissociate the preformed IMPα/β1 heterodimer, which is responsible for nuclear transport of viral protein cargos [49]. As nuclear transport of viral proteins is essential for the replication cycle and inhibition of the host’s antiviral response, targeting the nuclear transport process may be a viable therapeutic approach toward RNA viruses [50,51]. Recently, an in vivo study has proven Ivermectin’s capability to reduce viral RNA up to 5,000-fold after 48 h of infection with SARS-CoV-2 [52]. With an established safety profile for anti-parasitic use, the next step to prove Ivermectin’s efficacy on treating COVID-19 involves trials to figure out the adequate dosing.

#### 7.2.2. Lopinavir/Ritonavir

Aspartyl protease is an enzyme encoded by the *pol* gene of the human immunodeficiency virus (HIV) that cleaves the precursor polypeptides in HIV, thus playing an essential role in its replication cycle. The HIV protease inhibitors, lopinavir and ritonavir, are therefore used in combination as HIV therapeutic drugs. Although coronaviruses encode a different enzymatic class of protease, the cysteine protease, theoretical evidence exists that lopinavir and ritonavir also inhibit the coronaviral 3CL1^pro^ protease [53,54,55,56]. More importantly, a number of clinical, animal, and in vitro model studies performed on SARS and MERS proved it to be effective against the respective viruses [46,47,48,49]. Lopinavir/ritonavir combination was engaged in a clinical trial against COVID-19 in patients with mild and moderate COVID-19 (NCT04252885), however, it showed little benefit for improving the clinical outcome [45]. In another trial performed on patients with severe COVID-19 (ChiCTR2000029308), no benefits of lopinavir/ritonavir beyond standard care were observed [57].

### 7.3. Blocking Virus–Cell Membrane Fusion

#### 7.3.1. Recombinant Human Angiotensin-converting Enzyme 2 (APN01)

The soluble recombinant human Angiotensin-converting Enzyme 2 (rhACE2) is expected to block the entry of SARS-CoV-2 by blocking the S protein from interacting with the cellular ACE2. Indeed, in a recent study, it was reported that rhACE2 could inhibit SARS-CoV-2 replication in cellular and embryonic stem cell-derived organoids by a factor 1,000-5,000 times [58]. It is believed that the administration of the recombinant human Angiotensin-converting Enzyme 2 (rhACE2) can decrease serum level of angiotensin II by directing the substrate away from the related enzyme, ACE. Like mentioned earlier, this could prevent further activation of ACE2 receptor and thereby preserve the pulmonary vascular integrity and prevent ARDS [59]. APN01, originally developed by Apeiron Biologics, has already undergone phase II trial for ARDS. A small pilot study in China (NCT04287686) is now evaluating the biological and physiological role of rhACE2 in COVID-19 pneumonia, especially as a treatment of ARDS. Later, Apeiron Biologics has initiated a placebo controlled, double blinded, dose-escalation study to access the safety and tolerability of intravenous APN01. It is believed that by measuring plasma level of angiotensin II and angiotensin 1-7, the bioproducts interfered by the potential drug, the biological and physiological role of rhACE2 in COVID-19 pneumonia could be evaluated.

#### 7.3.2. Hydroxychloroquine

As a well-known antimalarial and anti-autoimmune agent, hydroxychloroquine can also block virus infection by increasing endosomal pH required for membrane fusion between the virus and the host cell [60]. Moreover, it was shown to specifically inhibit the replication of SARS-CoV by interfering with the glycosylation of its cellular receptor, ACE2 [61]. Recently, in vitro testing revealed its ability to effectively reduce the viral copy number of SARS-CoV-2 [24]. Therefore, a number of clinical trials have been quickly conducted in China, which demonstrated that hydroxychloroquine was to various degree effective in treatment of COVID-19-associated pneumonia. Similarly, in a small open-label non-randomized clinical trial from France, hydroxychloroquine demonstrated positive effect in combination with azithromycin [62]. In the wake of this evidence, the U.S. FDA issued an Emergency Use Authorization for the use of hydroxychloroquine to treat COVID-19 in the USA. It is noteworthy that the latest study found no evidence of clinical benefit of the combination of hydroxychloroquine and azithromycin for the treatment of 11 patients with severe COVID-19 [63], thus, larger randomized controlled trials are needed for further evaluation

#### 7.3.3. Arbidol Hydrochloride (Umifenovir)

Approved by Russia and China, Arbidol is an entry inhibitor against influenza viruses and arboviruses [64]. Targeting hemagglutinin (HA), the major glycoprotein on the surface of influenza virus, arbidol prevents the fusion of the viral membrane with the endosome after endocytosis. Currently, it is undergoing trials as a single agent (NCT04260594, NCT04255017). In a randomized clinical trial aimed at comparing arbidol with favipiravir (ChiCTR2000030254), the latter was demonstrated to be far more superior in treatment outcome [65].

### 7.4. Enhancing the Innate Immune System

#### 7.4.1. Natural Killer Cells

The highest mortality rate inflicted by COVID-19 is observed among the elderly patients, which may be explained by the weakening of the immune system with age. Therefore, the approaches aimed at boosting the innate anti-viral immune responses are of great potential. Natural killer (NK) cells constitute an important component of the innate immune system that ensures rapid response to viral infection. Previous study has shown that pulmonary migration of NK cells and macrophages plays a significant role in the clearance of SARS-CoV [66]. The innate response itself, without the assistance from the CD8+ T cells and antibodies, is able to control SARS-CoV infection by increasing the production of cytokines and chemokines. Whether the addition of NK cells could help to reach viral clearance in COVID-19 pneumonia is under phase I trial (NCT04280224) in China estimated to be completed by the end of 2020.

Several companies aim to repurpose their anti-cancer NK-based products to treat COVID-19. Among them are the jointly developed product from the Green Cross LabCell from South Korea with Kleo Pharmaceuticals from the U.S. The USA-based company Celularity has developed the placenta haematopoetic stem cell-derived NK cells, CYNK-001.

#### 7.4.2. Reombinant Interferon

Type I interferons are secreted by the virus-infected cells. When used alone or in combination with other drugs, they exert a broad-spectrum antiviral effect against HCV, respiratory syncytial virus, SARS-CoV [67] and MERS-CoV [68]. Trials are now focusing on their safety and efficacy in treating COVID-19 pneumonia (NCT04293887).

### 7.5. Attenuating the Inflammatory Response

#### 7.5.1. Mesenchymal Stem Cells

Mesenchymal stem cells (MSCs) have been proven to exert anti-inflammatory function by decreasing pro-inflammatory cytokines and producing paracrine factors to repair tissues. Preclinical evidence has also shown that MSCs are able not only to restore endothelial permeability, but also reduce inflammatory infiltrate [69]. While the immunomodulating effects of MSCs have been proven on avian influenza viruses [70], their role in COVID-19 pneumonia is still under evaluation. At present, MSCs from the umbilical cord and dental pulp are being tested (NCT04293692, NCT04269525, NCT04288102, NCT04302519).

#### 7.5.2. Intravenous Immunoglobulin

Intravenous immunoglobulin (IVIG) has been widely applied in the field of neurology, dermatology and rheumatology. In a dose-dependent manner, IVIG exerts diverse effects on the immune system. Generally, at low doses (0.2-0.4g/kg), IVIG is used as a replacement therapy for antibody deficiencies. While at higher doses (up to 2g/kg), IVIG exhibits its immunomodulatory functions, such as suppressing inflammatory cells proliferation, inhibiting phagocytosis and interfering antibody-dependent cytotoxicity [71]. Current trials focus on the supplementary effects of low dose IVIG (0.5g/kg for 5 days, NCT04261426).

#### 7.5.3. SARS-CoV-2-Specific Neutralizing Antibodies

The humoral immune response mediated by antibodies is crucial for preventing viral infections. Therefore, the development of the specific surface epitope-targeting neutralizing antibodies is a more long-term, albeit more specific approach to target COVID-19 [72]. AbCellera (Canada) and Eli Lilly and Company (USA) are co-developing a functional antibody that could neutralize SARS-CoV-2 in infected patients. For this purpose, they screened through more than 5 million immune cells from one of the first U.S. patients who recovered from COVID-19, and identified more than 500 of the potential anti-SARS-CoV-2 antibody sequences, which are currently undergoing screening to find the most effective ones. Whereas such an approach is time-consuming and effort-demanding, it is encouraging that it has been successfully applied to manufacture the specific functional antibodies against the West Nile virus [73].

Vir Biotechnology, Inc., ImmunoPrecise, Mount Sinai Health System, and Harbour BioMed (HBM) are also screening to find monoclonal antibodies to tackle SARS-CoV-2. Although all of them are still in preclinical stages, eligible candidates are expected to exert both prophylactic and therapeutic effects.

#### 7.5.4. Anti-C5a Monoclonal Antibody

As complement activation has been demonstrated in acute lung injury, C5a, the bioactive molecule cleaved from C5, is responsible for the full development of tissue injury. The role of C5a includes recruitment of neutrophils and T-lymphocytes, and increasing pulmonary vascular permeability [74]. It has also been proved that anti-C5a treatment could reduce lung injury by decreasing vascular leakage and neutrophil influx into the alveolar space. Hence, BDB-1, launched by Beijing Defengrei Biotechnology Co., and IFX-1, produced by Beijing Staidson Biopharma and InflaRx, are anti-C5a monoclonal antibodies targeting the foundation of the inflammatory response, expected to attenuate the level of lung injury caused by SARS-CoV-2.

#### 7.5.5. Blocking the Interleukin (IL)-6 Pathway

Interleukin (IL)-6, TNF-α, and IL-1 are the most important pro-inflammatory cytokines in the human body. Specifically, IL-6 is a predictive factor of poor prognosis in patients with ARDS [75]. Recently it has also been reported that the elevated interleukin-6 (IL-6) is strongly associated with the need for mechanical ventilation [76]. The classical pathway of IL-6 signaling occurs through IL-6 receptors, which are expressed on neutrophils, monocytes, macrophages, and other leukocyte populations [77]. Besides binding to the membrane-bound IL-6 receptor (mIL-6R, CD126), IL-6 can also bind to the soluble form of IL-6 receptor created by proteolytic cleavage of mIL-6R or alternative splicing of mRNA. An elevated level of circulating IL-6 is associated with a faster decline of lung elasticity and more severe bronchoalveolar inflammation. Hence, specific blockade of IL-6-regulated signaling pathways represents a promising approach to attenuate inflammation-associated damage [78]. Moreover, it is reported that in the tuberculosis model in mice, IL-6 blockade did not lead to an increase of bacterial burden in the lung. On the contrary, neutralizing TNFα antibodies resulted in a significant increase in mycobacterial colony units.

EUSA Pharma has initiated a study to evaluate the effectiveness of siltuximab, a monocloncal antibody against IL-6, in treating COVID-19 patients with ARDS. On the other hand, Tiziana Life Sciences is developing an anti-interleukin-6 receptor antibody, TZLS-501, to treat the disease from another perspective. The company believes that administrating anti-IL-6 antibody could result in an inhibition of downstream signaling either membrane bound or soluble IL-6 receptors, and a reduction in circulating IL-6 levels. Regeneron Pharmaceuticals and Sanofi are also conducting a phase II and a phase III trial on Kevzara (sarilumab), an IL-6 receptor antagonist, in severe and critical COVID-19 patients (NCT04315298). Though Kevzara has proven its efficacy in treating arthritis [79], its efficacy in treating SARS-CoV-2 is still unknown.

#### 7.5.6. Thalidomide

Recently, thalidomide has re-emerged as an antiangiogenic, anti-inflammatory, and anti-fibrotic agent. Through decreasing the synthesis of TNF-alpha, thalidomide has been used as a treatment for multiple inflammatory diseases, such as Crohns disease and Behcets disease [80]. In addition, preclinical studies proved that thalidomide was effective in treating H1N1-infected mice by reducing infiltration of inflammatory cells and the production of pro-inflammatory cytokines [81]. Current studies are focusing on its immunomodulatory effects that could lessen lung injury caused by excessive immune response to SARS-CoV-2 (NCT04273529, NCT04273581).

#### 7.5.7. Methylprednisolone

As was mentioned earlier, systemic glucocorticoids are currently contraindicated in SARS-CoV-2 infection, as they may prolong viral clearance. However, it is also known that the underlying pathogenesis of COVID-19 pneumonia is composed of both the direct damage caused by the virus and the excessive immune response from the host. Thus, whether methylprednisolone administration would help suppress the unwanted immune reactions is controversial, therefore, studies have been initiated to explore its effectiveness and safety (NCT04273321, NCT04263402).

#### 7.5.8. Fingolimod

Fingolimod is an oral immunomodulating agent that is primarily used to treat refractory multiple sclerosis. By structurally resembling the lipid sphingosine-1-phosphate (S1P), fingolimod can act as a highly potent functional antagonist of S1P1 receptors in the lymph node T cells. Through the effective binding, S1P1 receptors are internalized and the lymph node T cells are subsequently sequestered [82]. Decreased pulmonary influx of T lymphocytes is another approach to attenuate uncontrolled immunopathogenesis (NCT04280588).

### 7.6. Symptomatic Control

#### Bevacizumab

Elevated vascular endothelial growth factor (VEGF) level is observed in patients with acute respiratory distress syndrome. VEGF functions as a mediator that can induce endothelial injury and increase microvascular permeability [83]. Bevacizumab, a recombinant humanized monoclonal antibody widely used in treating multiple types of cancers, is capable of blocking angiogenesis by specific binding to VEGF. An ongoing trial is now evaluating the effectiveness of Bevacizumab as a unique approach to treat SARS-CoV-2 infection (NCT04275414).

### 7.7. Vaccine

The development of vaccine represents a more long-term strategy to prevent COVID-19 outbreaks in the future. With the sequencing of SARS-CoV-2 genome, multiple nucleic acid-based vaccine candidates have been proposed, mostly based on the S protein-coding sequence.

#### 7.7.1. mRNA-1273

In early January 2020, soon after the outbreak of COVID-19 pneumonia, the genome of SARS-CoV-2 has been sequenced. Moderna’s mRNA-1273 is a synthetic strand of mRNA that encodes the prefusion-stabilized viral spike protein. After intramuscular injection to human bodies, it is expected to elicit antiviral response specifically toward the spike protein of SARS-CoV-2. Besides, unlike conventional vaccines, which are either made from inactivated pathogen or small subunits of live pathogen, synthesis of the lipid nanoparticle-encapsulated mRNA vaccine does not require the virus. Therefore, it is relatively safe and ready to be tested. If mRNA-1273 proves to be safe for humans and pass the phase I trial, successive evaluation of its efficacy will be carried out immediately (NCT04283461).

#### 7.7.2. INO-4800

INO-4800 is a DNA vaccine candidate created by Inovio Pharmaceuticals. Like Moderna’s mRNA-1273, INO-4800 is also a genetic vaccine that can be delivered to human cells and translated into proteins to elicit immune responses. Compared to conventional vaccines, genetic vaccines require lower costs of production and easier way of purification. The simple structure of nucleic acids also obviates the risk of incorrect folding, which could occur in recombinant protein-based vaccines [68,69]. However, the amount of plasmid delivered and the adequate interval and route of administration are the factors that may influence the immunogenicity of genetic vaccines.

#### 7.7.3. ChAdOx1 nCoV-19

This vaccine, created by the University of Oxford, is composed of a non-replicating adenovirus vector and the genetic sequence of the S protein of SARS-CoV-2, and has entered a phase I/II clinical trial (NCT04324606). The non-replicating nature of adenovirus in the host makes it relatively safe in children and individuals with underlying diseases. Besides, the adenovirus-based vectors are characterized by a broad range of tissue tropism that covers both respiratory and gastrointestinal epithelium, the two main sites that express the ACE-2 receptor of SARS-CoV-2. However, the possibility of dominant immunogenicity toward the vector genes rather than the transgenes should always be considered [45].

#### 7.7.4. Stabilized Subunit Vaccines

Enveloped viruses require fusion of the viral membrane with the host cell membrane for infection. This process involves the conformational change of the viral glycoprotein from the pre-fusion form to the post-fusion form. Although the pre-fusion glycoproteins are relatively unstable, they are still able to elicit strong immune responses [84]. Thus, the University of Queensland is developing a stabilized subunit vaccine based on the molecular clamp technology, which would allow recombinant viral proteins to stably remain in their pre-fusion form. Previously applied to influenza virus and Ebola virus, molecular clamp vaccines have proved their capacity to induce the production of neutralizing antibodies. They were also reported to be potent after two weeks at 37 °C.

#### 7.7.5. Nanoparticle-Based Vaccines

Nanoparticle-based platforms represent an alternative strategy to incorporate antigens. Through encapsulation or covalent functionalization, nanoparticles can be conjugated with antigenic epitopes, mimic viruses and provoke antigen-specific lymphocyte proliferation as well as cytokine production. In addition, mucosal vaccination through intranasal or oral spray can not only stimulate immune reactions at the mucosal surface, but also provoke systemic responses [85]. This demonstrates the potential of nanoparticle-based vaccines to protect humans against respiratory viruses that cause systemic symptoms. Novavax, Inc. is producing a nanoparticle-based vaccine using antigens derived from the coronavirus S protein. The protein is stably expressed in the baculovirus system, and the product is anticipated to enter phase I trial this summer.

### 7.8. Pathogen-Specific Artificial Antigen-Presenting Cells

Based on the knowledge that antigen-specific T cells are able to eradicate cancer cells as well as viral infections, generating large amounts of T cells with viral antigen specificity in a timely manner may well help us withstand the invasion of SARS-CoV-2. Efficient methods to produce massive amounts of T cells include appropriate antigen-presenting cells that can activate effector T cells, and the differentiation and proliferation of corresponding effector, cytotoxic T cells [24].

Hence, the genetically modified artificial antigen-presenting cells (aAPCs) that express the conserved domains of the viral structural proteins delivered by lentivirus vector are supposed to evoke the naïve T cells in the human body and lead to differentiation and proliferation. Trials are now evaluating the safety and immunogenicity of aAPCs alone and in combination with antigen-specific cytotoxic T cells (NCT04299724, NCT04276896).

## 8. Conclusions

In this article, we present an overview of the current state of knowledge on the SARS-CoV-2 and COVID-19 pandemic. In addition to an overview of the epidemiological, clinical, and radiological features of SARS-CoV-2 (Figure 1), we also summarize possible therapeutic options currently under investigation and the future outlook for the disease. We also speculate on several mechanisms contributing to the novel profile of COVID-19 pneumonia, including its high transmissibility caused by unvaried ACE2 structure at the viral binding site among different populations, and its unique progression pattern, in which patients could be asymptomatic, yet presenting positive radiographic or laboratory findings.

We summarize the current clinical trials that have been rapidly initiated upon the onset of the pandemic emergency and are currently undergoing as for April 2020. Most of them are based on repurposing the therapeutic agents previously designed for other applications. These agents can be divided into two broad categories, those that can directly target the virus replication cycle, and those based on immunotherapy approaches either aimed to boost innate antiviral immune responses or alleviate damage induced by dysregulated inflammatory responses. Whereas the vaccines and therapeutic antibodies aimed to specifically target SARS-CoV-2 are also being tested, this solution is more long-term, as they require thorough testing of their safety. On the other hand, drug repurposing happens to be the only practical approach as a rapid response measure to the emergent pandemic, as most of these agents have already been tested for their safety, and have demonstrated their action against other viruses, including the related SARS-CoV and MERS-CoV. These drugs target different viral infection response pathways, or directly interfere with the virus replication cycle as summarized in Figure 2. As for now, some of them have already demonstrated promising results in the initial clinical trials performed in the wake of the pandemic and have been approved for a wider use. The promising examples include *favipiravir*, an influenza drug that interferes with the viral replication, and *hydroxychloroquine*, a repurposed antimalarial drug that interferes with the virus endosomal entry pathway.

By posing a significant challenge toward the public health system and the existing antiviral strategies, SARS-CoV-2 has undoubtedly grabbed the globe’s attention in the beginning of 2020. We believe this may be a trigger for more systematic approaches to prepare ourselves in advance for any potential future pandemics. In the era of big data, more systematic approaches to identify the potential agents for drug repurposing can be applied. Noteworthy is the recent study by Zhou et al., whereby an integrative analysis of interactome networks associated with human coronaviruses and drugs targeting the components of these networks was applied to reveal 16 novel candidates for drug repurposing [86].

We believe that such computational techniques combined with the follow-up experimental studies aimed to test the computationally predicted antiviral agents can help us to have a wider arsenal of potentially repurposed drugs in the case of any future virus outbreaks.

## Figures and Tables

**Table 1:** Comparison of the epidemiological, clinical and radiological features of the diseases caused by SARS-CoV, MERS-CoV, and SARS-CoV-2.

	SARS-CoV	MERS-CoV	SARS-CoV-2
Disease	SARS	MERS	COVID-19
**Transmission**	Respiratory droplets Close contact with diseased patients Fecal-oral Aerosol [26]	Respiratory droplets Close contact with diseased patients/camels Ingestion of camel milk	Respiratory droplets Close contact with diseased patients Possibly fecal-oral [7] Possibly aerosol [27]
**Latency**	2–7 days	2–14 days	97.5% became symptomatic within 11.5 days (CI, 8.2 to 15.6 days) [28]
**Contagious period**	10 days after onset of disease	When virus could be isolated from infected patients	Unknown
**Reservoir**	Bats	Bats	Bats
**Incidental host**	Masked palm civets	Dromedary camels	Malayan pangolin [29]
**Origin**	Guangdong, China	Saudi Arabia	Hubei, China
**Fatality rate**	~10%	~36%	~2.3%
**Radiologic features**	Diverse from focal faint patchy ground-glass opacities to bilateral ill-defined air space consolidations on plain chest radiograph. Non-specific to distinguish between three different diseases. Ref. [30,31,32,33]		
**Clinical presentation**	From asymptomatic or mild disease to acute upper respiratory distress and multiorgan failure leading to death. Varies between individuals. Ref. [34] Vomiting and diarrhea are also reported.		
